# Platelets are activated in ANCA-associated vasculitis *via* thrombin-PARs pathway and can activate the alternative complement pathway

**DOI:** 10.1186/s13075-017-1458-y

**Published:** 2017-11-15

**Authors:** Di Miao, Dan-Yang Li, Min Chen, Ming-Hui Zhao

**Affiliations:** 1 Renal Division, Department of Medicine, Peking University First Hospital, Beijing, 100034 China; 2 Institute of Nephrology, Peking University, Beijing, 100034 China; 3 Key Laboratory of Renal Disease, Ministry of Health of China, Beijing, 100034 China; 4 Key Laboratory of Chronic Kidney Disease Prevention and Treatment, Ministry of Education, Peking University, Beijing, 100034 China; 5 Peking-Tsinghua Center for Life Sciences, Peking University, Beijing, 100871 China

**Keywords:** ANCA, Vasculitis, Platelets, Thrombin, Protease-activated receptors, Complement

## Abstract

**Background:**

In this study, we investigated the mechanism of platelet activation in patients with antineutrophil cytoplasmic antibody (ANCA)-associated vasculitis (AAV), as well as the activation of the alternative complement pathway by platelets in AAV.

**Methods:**

CD62P and platelet-leukocyte aggregates in AAV patients were tested by flow cytometry. Platelets were stimulated by plasma from active AAV patients. The effect of the thrombin-protease-activated receptors (PARs) pathway was evaluated by blocking thrombin or PAR1 antagonists. After platelets were activated by plasma from AAV patients, Ca/Mg-Tyrode’s buffer and Mg-EGTA buffer were used to measure complement activation in liquid phase and on the surface of platelets.

**Results:**

The levels of CD62P-expressing platelets and platelet-leukocyte aggregates were significantly higher in active AAV patients than those in remission and normal controls. Platelets were activated by plasma from active AAV patients (percentage of CD62P-expressing platelets, 97.7 ± 3% vs. 1 ± 0.2%, *p* < 0.0001, compared with those incubated with healthy donor plasma), and this was inhibited by thrombin or PAR1 antagonists (percentage of CD62P-expressing platelets, 97.7 ± 3% vs*.* 2.7 ± 1%, *p* < 0.0001, 97.7 ± 3% vs. 5 ± 1.4%, *p* < 0.0001, respectively). Platelets activated by plasma from AAV patients could trigger complement activation *via* the alternative pathway, as demonstrated by significant elevation of C3a, C5a, and sC5b-9 and significantly more C3c and C5b-9 deposition on the surface of platelets.

**Conclusions:**

Platelets were activated in AAV patients, and such activation was at least partially attributed to the thrombin-PARs pathway. Activated platelets triggered the alternative complement pathway in AAV.

## Background

Antineutrophil cytoplasmic antibody (ANCA)-associated vasculitis (AAV) comprises a group of autoimmune diseases that mainly affect small vessels, including microscopic polyangiitis (MPA), granulomatosis with polyangiitis (GPA) and eosinophilic granulomatosis with polyangiitis (EGPA). The two major antigens in ANCA are myeloperoxidase (MPO) and proteinase 3 (PR3). Cumulative evidence suggests that complement system activation *via* the alternative pathway is indispensable for the development of AAV [1–3]. The mechanism of alternative complement pathway activation in AAV is not fully understood, and it is mainly considered to be a downstream effect of neutrophil activation [1, 4, 5].

Increasing studies demonstrate a high prevalence of venous thromboembolic event (VTE) and a hypercoagulant state in AAV patients [6–11]. In the active stage of AAV, the platelet count is usually elevated [10, 12]. In addition to their classic hemostatic role, platelets are also inflammation protagonists [13–16]. Platelets and the soluble molecules that they secrete can mediate inflammatory responses and thus contribute to vascular injury [17, 18]. An association between some platelet indices, including platelet counts, mean platelet volume, and disease activity have been reported in AAV and several other autoimmune diseases, such as inflammatory bowel disease, ankylosing spondylitis and rheumatoid arthritis [10, 12, 19–21].

Thrombin, the main effector protease in the coagulation cascade, is one of the most potent platelet activators [22, 23]. Activation by thrombin initiates platelet degranulation and secretion, which translocates adhesion receptors to the cell surface, and releases hemostatic and inflammatory agonists/mediators into circulation, then causes surface molecule expression facilitating cellular adhesion [24]. Platelet activation by thrombin depends, at least in part, on signal transduction mediated by a family of G protein-coupled protease-activated receptors (PARs) in many diseases.

Our recent study demonstrated that the treatment of C5a-primed neutrophils with ANCA resulted in the release of tissue factor (TF), which subsequently led to thrombin generation [11]. We hypothesize that platelets are activated in AAV patients *via* the thrombin-PARs pathway, and such activated platelets can subsequently activate the alternative complement pathway in AAV.

## Methods

### Detection of platelet activation by flow cytometry

We analyzed the platelet activation parameters in AAV patients by flow cytometry, including CD62P and platelet adhesion to neutrophils, lymphocytes, and monocytes.

Blood samples were freshly collected from 31 patients with active AAV before the initiation of immunosuppressive therapy. Blood samples of 27 patients with AAV, who achieved complete remission at least 3–6 months after the initiation of immunosuppressive therapy, were also collected at their regular ambulatory visits. All the AAV patients in the current study met the 2012 revised Chapel Hill Consensus Conference criteria for AAV [25]. Disease activity was evaluated by the Birmingham Vasculitis Activity Score (BVAS) [26]. “Remission” was defined as “absence of disease activity attributable to active disease qualified by the need for ongoing stable maintenance immunosuppressive therapy” (complete remission), or “at least 50% reduction of disease activity score and absence of new manifestations” (partial remission) [27]. The BVAS levels of all the AAV patients in remission stage in this study were 0.

Treatment protocols have been described previously [28, 29]. In brief, induction therapy included corticosteroids in combination with cyclophosphamide (CTX). Oral prednisone was prescribed at an initial dosage of 1 mg/kg/day for 4–6 weeks, with reducing doses over time to 12.5–15 mg by 3 months. CTX was administered by daily oral dose of 2 mg/kg/day or intravenously 0.5 g/m^2^ every month. Patients with acute renal failure or pulmonary hemorrhage received three pulses of intravenous methylprednisolone (7–15 mg/kg/day) before the standard induction therapy. Patients with severe pulmonary hemorrhage or acute renal failure requiring dialysis at diagnosis received additional plasma exchanges. For maintenance therapy, daily oral azathioprine (AZA) was given (2 mg/kg/day) for at least 2 years.

Additionally, the blood samples of 40 healthy blood donors were also collected as normal controls. No VTE was observed by the time of blood collection, and none of the patients or healthy donors was receiving anticoagulant or antiplatelet therapies.

Citrate anticoagulated blood from patients and healthy donors were dispensed into an antibody cocktail containing CD41a APC (BD Pharmingen, San Jose, CA, USA), CD62P BV421 (BD Horizon™) and CD45-APC-H7 (BD Pharmingen) incubated for 15 min and analyzed by flow cytometry. CD41a-APC was used to gate platelets. As CD62P is a typical marker of platelet α-granule degranulation and lysosome degranulation, it serves as a marker of platelet activation [30–32]. Neutrophils, lymphocytes and monocytes were identified based on their characteristic light scatter, and differential CD45 expression [33].

### Isolation of platelets

Platelets were isolated according to the methods described previously [30]. Briefly, blood was drawn from healthy donors, using a 21G or larger bore needle and a light tourniquet, into a sodium citrate vacutainer. Platelet-rich plasma (PRP) was formed by centrifugation at 200 × g for 15 min and then washed using a citrate wash buffer (11 mM glucose, 128 mM NaCl, 4.3 mM NaH_2_PO_4_, 7.5 mM Na_2_HPO_4_, 4.8 mM Na_3_C_6_H_5_O_7_, 2.4 mM C_6_H_8_O_7_, 0.35% BSA, 50 ng/mL prostaglandin PGE1, pH 6.5) [30]. Platelets were pelleted by centrifugation at 1200 × g for 10 min and resuspended in modified Tyrode’s buffer (Leagene), as previously described [34].

### Stimulation and inhibition assays

Heparinized plasma samples from 14 active AAV patients and 14 patients with minimal change disease (MCD, as the disease control) were collected immediately after centrifugation at 2000 g for 15 minutes at 4 °C. Then, the plasma was aliquoted and stored at −80 °C until use. Repeated freeze/thaw cycles were avoided.

Platelets that were resuspended in modified Tyrode’s buffer were incubated at 37 °C for 15 min with plasma from patients at a final concentration of 50%. This concentration was chosen because it demonstrated the highest platelet activation in our pilot experiments. According to the previous study, 1 mM Gly-Pro-Arg-Pro (GPRP, Sigma-Aldrich, St. Louis, MO, USA) was added to prevent fibrin polymerization [35]. To investigate the potential role of the thrombin-PARs pathway in platelet activation by plasma from AAV patients, plasma was pretreated with 20 μM D-Phe-Pro-Arg-CH_2_Cl (PPACK, EMD Millipore, Billerica, MS, USA), a thrombin inhibitor, and the cells were pretreated with 200 μM of a PAR1 inhibitor, SCH79797 (C_23_H_25_N_5_.2HCl, Abcam, Cambridge, MA, USA), as previously described [36, 37]. Thrombin receptor-activating peptide (TRAP, Sigma-Aldrich), a platelet agonist, was used as a positive control for platelet activation [24].

### Alternative complement pathway activation

Detection of complement activation was conducted according to the methods described previously [34, 38]. Briefly, non-activated or activated platelets were washed once with Tyrode/PGE buffer (Tyrode’s buffer with 1 μM PGE1 obtained from Sigma-Aldrich), centrifuged at 1200 g for 10 min at room temperature, and incubated with 50% plasma in 0.8 mM Mg-EGTA buffer (10 mM EGTA with 0.8 mM MgCl_2_) to assess complement proteins activation. Instead of reaction buffers, 10 mM EDTA was added, which binds calcium and magnesium ions in the plasma, to restrain potential complement activation as the negative control. The samples were incubated for 60 min at 37 °C, and then the reactions were stopped by 3 μl EDTA/PGE buffer (EDTA buffer with 1 μM PGE1). After centrifugation at 1200 g for 10 min at room temperature, the supernatant was stored and quantitated with Quidel enzyme-linked immunosorbent assays (ELISAs) for human C3a, C5a, sC4d, and sC5b-9 (Quidel Corporation, San Diego, CA, USA). The remaining platelets were washed with Tyrode/PGE buffer to reduce nonspecific adhesion of complement molecules, then incubated with antibodies to CD41, C3c and C5b-9, and analyzed by fluorescent-activated cell sorting (FACS), as described above.

The study protocol complied with the Declaration of Helsinki and was approved by the ethics committee of Peking University First Hospital. Written informed consent was obtained from each participant.

### Statistical analysis

Results were presented as the mean ± SD (for normally distributed data) or median and interquartile range (IQR, for non-normally distributed data), as appropriate. Difference in the paired samples was assessed using paired *t* test or Wilcoxon’s signed rank tests, as appropriate. Difference between unpaired groups was assessed using *t* tests or nonparametric tests, as appropriate. Difference between multiple groups was assessed using the one-way ANOVA. Difference between non-independent data was assessed using generalized estimating equations. Statistical analyses were performed with the SPSS statistics software, version 15.0 (Chicago, IL, USA). The *p* values less than 0.05 were considered significant.

## Results

### Platelets are activated in active AAV patients

Platelets from the patients and healthy donors were stained for CD62P. Among the 31 active AAV patient samples that were analyzed for CD62P expression, 19 were male and 12 were female, with an age of 59.4 ± 13.2 years at diagnosis. Twenty-seven and four patients were MPO-ANCA and PR3-ANCA positive, respectively. The initial serum creatinine (Scr) values were 456.8 ± 258.6 μmol/L. The initial BVAS levels were 19.5 ± 6.2 (Table 1).

**Table 1:** General data of the 31 AAV patients in active stage

Parameters	Value
General clinical data	
No. subjects	31
Gender (M/F)	19/12
Age	59.4 ± 13.2
ANCA target antigen (MPO/PR3)	29/3
Initial Scr (μmol/L)	448.6 ± 250.6
Urinary protein (g/24 hr)	0.76 (00.5–2.01)
BVAS	18.3 ± 5.7
Renal involvement	21 (91.3%)
Pulmonary involvement	21 (91.3%)
ENT involvement	6 (26.1%)
Nervous system involvement	5 (21.7%)
Pathologic data	
No. subjects	20
Glomerular lesions	
Total crescents	58% (36–77%)
Cellular crescents	42% (26–57%)
Tubulointerstitial lesions	
Interstitial infiltration(−/+/++/+++)	0/6/14/0
Interstitial fibrosis(−/+/++)	1/6/13
Tubular atrophy(−/+/++)	2/13/5
Treatment data	
Prednisone	31
Methylprednisolone pulse	17
Plasma exchange	15
CTX (intravenous/oral)	28/3

Abbreviations: *AAV* antineutrophil cytoplasmic antibody-associated vasculitis, *ANCA* antineutrophil cytoplasmic antibody, *MPO* myeloperoxidase, *PR3* protinase 3, *Scr* serum creatinine, *BVAS* Birmingham Vasculitis Activity Score, *ENT* ear, nose and throat, *CTX* cyclophosphamide

**Fig. 1:**
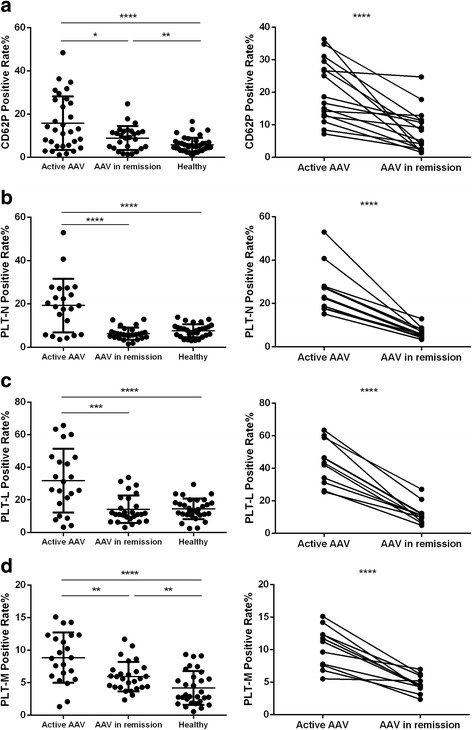
Platelet activation profiles and leukocyte-platelet aggregates in patients with ANCA-associated vasculitis. **a** CD62P-expressing platelets. **b** Neutrophil-platelet aggregation. **c** Lymphocyte-platelet aggregation. **d** Monocyte-platelet aggregation. *p < 0.05, **p < 0.01, ***p < 0.001, ****p < 0.0001, unpaired and paired *t* tests

The percentages of CD62P-expressing platelets in AAV patients in active stage were significantly higher than that in remission and the normal controls (15.8% [5.1%, 26.7%] vs*.* 8.9% [4.2%, 12%], *p* < 0.05; 15.8% [5.1%, 26.7%] vs*.* 5.7% [3.1%, 6.5%], *p* < 0.0001, respectively). Furthermore, among the 16 AAV patients with sequential blood samples from both active stage and remission, the percentages of CD62P-expressing platelets were significantly higher in AAV patients in active stage than those in remission (20.4% [12.7%, 28.9%] vs*.* 8.0% [2.5%,11.4%], *p* < 0.0001), and all these 16 patients had a decrease in the percentages of CD62P-expressing platelets in remission, compared with that in active stage (Fig. 1a), which was consistent with the above-mentioned results. There was no significant difference of the percentages of CD62P-expressing platelets among different induction therapy regimens in AAV patients.

Leukocytes from AAV patients and healthy blood donors were stained for CD45 and CD41a (the marker of platelets) in 22 of the above-mentioned 31 active AAV patients with a sufficient sample. Neutrophils, lymphocytes and monocytes were identified based on their characteristic light scatter and differential CD45 expression. The percentages of CD41a-positive neutrophils, lymphocytes and monocytes, i.e., neutrophil-, lymphocyte- and monocyte-platelet aggregates (abbreviated as NPA, LPA and MPA, respectively) were analyzed separately. The NPA, LPA and MPA percentages in the AAV patients in the active stage were significantly higher than those in the AAV patients in remission (19.3% [5.8%,27.2%] vs*.* 6.3% [4.6%, 7.2%], *p* < 0.0001; 31.8% [15.8%, 46.3%] vs*.* 14.2% [8.4%, 20.9%], *p* < 0.001; 8.8% [5.9%, 12.3%] vs. 5.9% [4.3%, 7%], *p* < 0.01; respectively) and in the normal controls (19.3% [5.8%,27.2%] vs*.* 7.7% [5.4%,9.5%], *p* < 0.0001; 31.8% [15.8%, 46.3%] vs*.* 14.5% [10.8%, 18.7%], *p* < 0.0001; 8.8% [5.9%, 12.3%] vs*.* 4.2% [2.1%, 6.1%], *p* < 0.0001; respectively) (Fig. 1b–d). Furthermore, the platelet-leukocyte aggregate levels in 11 AAV patients with sequential samples of both active stage and remission were compared. The neutrophil, lymphocyte and monocyte adhesion percentages were significantly higher in the active stage than in remission (26.4% [17.8%, 27.9%] vs*.* 6.6% [4.8%, 8.1%], *p* < 0.0001; 43.4% [31.2%, 58.9%] vs. 12% [7%,12.3%], *p* < 0.0001; 10.4% [7.6%, 12.3%] vs. 4.7% [4.1%,6%], *p* < 0.0001; respectively); all 11 patients had decreased NPA, LPA and MPA levels in remission compared with the active stage (Fig. 1b–d).

Collectively, these data suggest that platelets are activated in patients with active AAV.

### Effect of the thrombin-PARs pathway in platelet activation by plasma from AAV patients

**Fig. 2:**
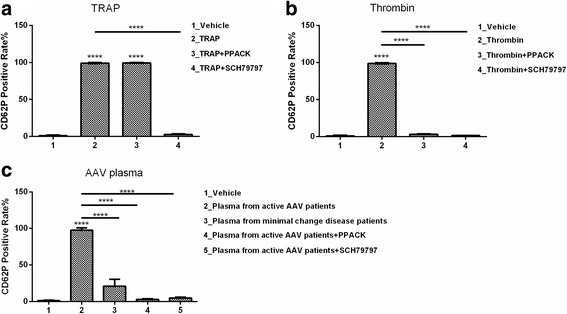
Effect of the thrombin-PARs pathway on platelet activation in AAV. **a** CD62P expression on platelets activated by TRAP. **b** CD62P expression on platelets activated by thrombin. **c** CD62P expression on platelets activated by AAV plasma. ****p < 0.0001, unpaired *t* test

We collected plasma samples from 14 active AAV patients before the initiation of immunosuppressive therapy for platelets stimulation. Among these 14 patients, 7 were male and 7 were female, with an average age of 52.0 ± 13.0 years at diagnosis. Seven and seven patients were MPO-ANCA and PR3-ANCA positive, respectively. The initial BVAS level was 20.6 ± 7.1. When the platelets were incubated with plasma from the active AAV patients, the CD62P-expressing platelet levels increased significantly compared with those incubated with plasma from the healthy donors (97.7 ± 3% vs*.* 1 ± 0.2%, *p* < 0.0001) (Fig. 2).

To evaluate the potential role of the thrombin-PARs pathway in platelet activation, thrombin inhibition and PAR1 blockage were performed. The plasma from active AAV patient was pretreated with 20 μM PPACK to inhibit its potential thrombin activity. PPACK pretreatment led to a significant decrease in the CD62P-expressing platelets after stimulation with plasma of AAV patients (97.7 ± 3.0% vs*.* 2.7 ± 1.0%, *p* < 0.0001). Furthermore, pre-incubation with 200 μM SCH79797 induced a significant decrease in the CD62P expression level after stimulation with plasma of AAV patients (97.7 ± 3.0% vs*.* 5 ± 1.4%, *p* < 0.0001) (Fig. 2).

Collectively, these results suggest that thrombin-PARs pathway contributes at least partially to platelet activation in AAV patients.

### Alternative complement pathway activation by AAV plasma-stimulated platelets

**Fig. 3:**
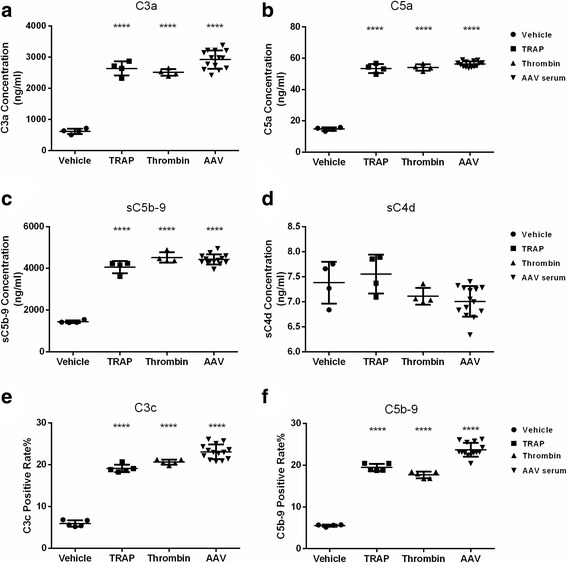
In Tyrode’s buffer, the complement system was activated by AAV-plasma stimulated platelets. **a** Generation of C3a by activated platelets. **b** Generation of C5a by activated platelets. **c** Generation of sC5b-9 by activated platelets. **d** Generation of sC4d by activated platelets. **e** Deposition of C3c on activated platelets. **f** Deposition of C5b-9 on activated platelets. ****p < 0.0001, compared with the vehicle group (unpaired *t* test)

After the platelets were activated by the plasma of AAV patients, Ca/Mg-Tyrode buffer was used to measure complement activation *via* all three pathways, i.e., the classical, mannose-binding lectin (MBL) and alternative pathways. In the liquid phase, the complement fragments C3a, C5a and soluble membrane attack complex (MAC) sC5b-9 levels were significantly higher than those in the vehicle treatment (2,921.5 ± 295.7 ng/ml vs*.* 618.1 ± 87.6 ng/ml, *p* < 0.0001; 56.3 ± 1.8 ng/ml vs. 14.8 ± 1.0 ng/ml, *p* < 0.0001; 4425.3 ± 243.8 ng/ml vs. 1445 ± 67.2 ng/ml, *p* < 0.0001; respectively), whereas sC4d, a common factor of the classical and mannose-binding lectin pathways, was comparable to that in the vehicle group (7.0 ± 0.3 ng/ml vs. 7.4 ± 0.4 ng/ml, *p* = 0.629). On the platelets, levels of adhesion complement fragment C3c, a cleavage product of C3b and the C5b-9 complex, were significantly higher than in the negative control samples (23.1 ± 1.7% vs. 5.9 ± 0.8%, *p* < 0.0001; 22.7 ± 1.6% vs. 5.9 ± 0.8%, *p* < 0.0001; respectively) (Fig. 3).

**Fig. 4:**
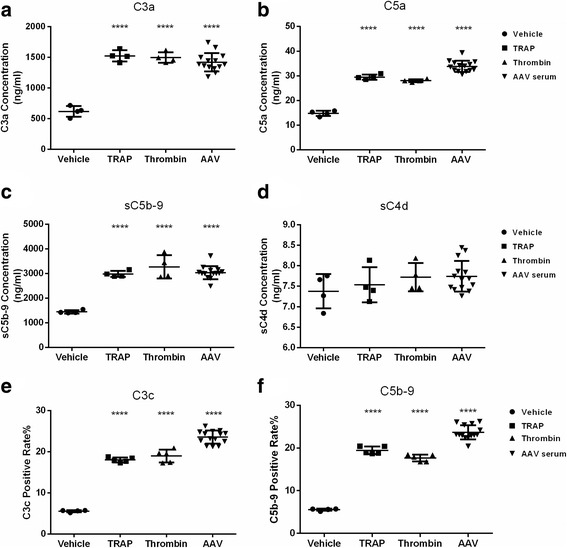
In EGTA buffer, the alternative pathway of complement system was activated by AAV-plasma stimulated platelets. **a** Generation of C3a by activated platelets. **b** Generation of C5a by activated platelets. **c** Generation of sC5b-9 by activated platelets. **d** Generation of sC4d by activated platelets. **e** Deposition of C3c on activated platelets. **f** Deposition of C5b-9 on activated platelets. ****p < 0.0001, compared with the vehicle group (unpaired *t* test)

Additionally, after platelets were activated by ANCA-positive plasma, Mg-EGTA buffer was used to selectively measure activation of the complement alternative pathway and not the classic or MBL pathways. In the fluid phase, the concentrations of the complement fragments, C3a, C5a and sC5b-9, were significantly higher than those in the vehicle samples (1420.5 ± 150.0 ng/ml vs. 618.1 ± 87.6 ng/ml, *p* < 0.0001; 33.8 ± 2.3 ng/ml vs. 14.8 ± 1 ng/ml, *p* < 0.0001; 3035.5 ± 267.6 ng/ml vs. 1445 ± 67.2 ng/ml, *p* < 0.0001; respectively). On the platelet surfaces, the C3c and C5b-9 complex levels were significantly higher than in the negative control samples (23.5 ± 1.6% vs. 5.5 ± 0.3%, *p* < 0.0001; 23.7 ± 1.7% vs. 5.5 ± 0.3%, *p* < 0.0001; respectively) (Fig. 4).

These results show that the alternative complement pathway is activated by activated platelets in AAV.

## Discussion

Platelets are linked to the pathogenesis of many autoimmune diseases. Recently, Tomasson et al. reported soluble P-selectin (CD62P) as a disease activity marker of AAV [19], and Willeke et al. reported increased platelet counts in active AAV [12]. Our previous study also found that active AAV patients had elevated platelet counts in peripheral blood [10].

To the best of our knowledge, this is the first study to demonstrate that active AAV patient plasma can activate platelets. This activation is, at least partially, mediated by the thrombin-PARs pathway. Many studies suggested a hypercoagulable state in AAV patients [1–6]. Our recent study found that C5a, in combination with ANCA, can stimulate neutrophil to release tissue factor and thus lead to thrombin generation [11]. Thus, it is reasonable to assume a higher thrombin level in the circulation of AAV patients [10, 11]. Thrombin inhibition and PARs blockade resulted in a significantly lower percentage of CD62P-expressing platelets, indicating that the thrombin-PARs pathway plays an important role in platelet activation in AAV. A variety of studies reported that upon activation, platelets produce and release stored cytokines, chemokines and polyphosphates to recruit leukocytes and progenitor cells to inflammation sites. Additionally, they express adhesion molecules that interact with other immune cells, and they release pro-inflammatory, anti-inflammatory, angiogenic and microparticle mediators into the circulation to orchestrate immune responses [13–16]. Our findings may potentiate platelets as an inflammatory effector cell in the pathogenesis of AAV.

Another important finding in our study is that platelets, after being activated by the plasma of AAV patients, are sufficient to activate the alternative pathway of complement system in the fluid phase as well as on the surface of platelets. Activation of the complement alternative pathway is a crucial aspect in the pathogenesis of AAV. The alternative pathway aggravated several inflammatory cytokines to augment and sustain acute inflammation in AAV and may be a pathogenic mechanism. It was previously considered that in AAV, complement alternative pathway activation resulted from neutrophil activation [1, 4, 5]. The current study provides another novel mechanism for alternative complement pathway activation in AAV.

**Fig. 5:**
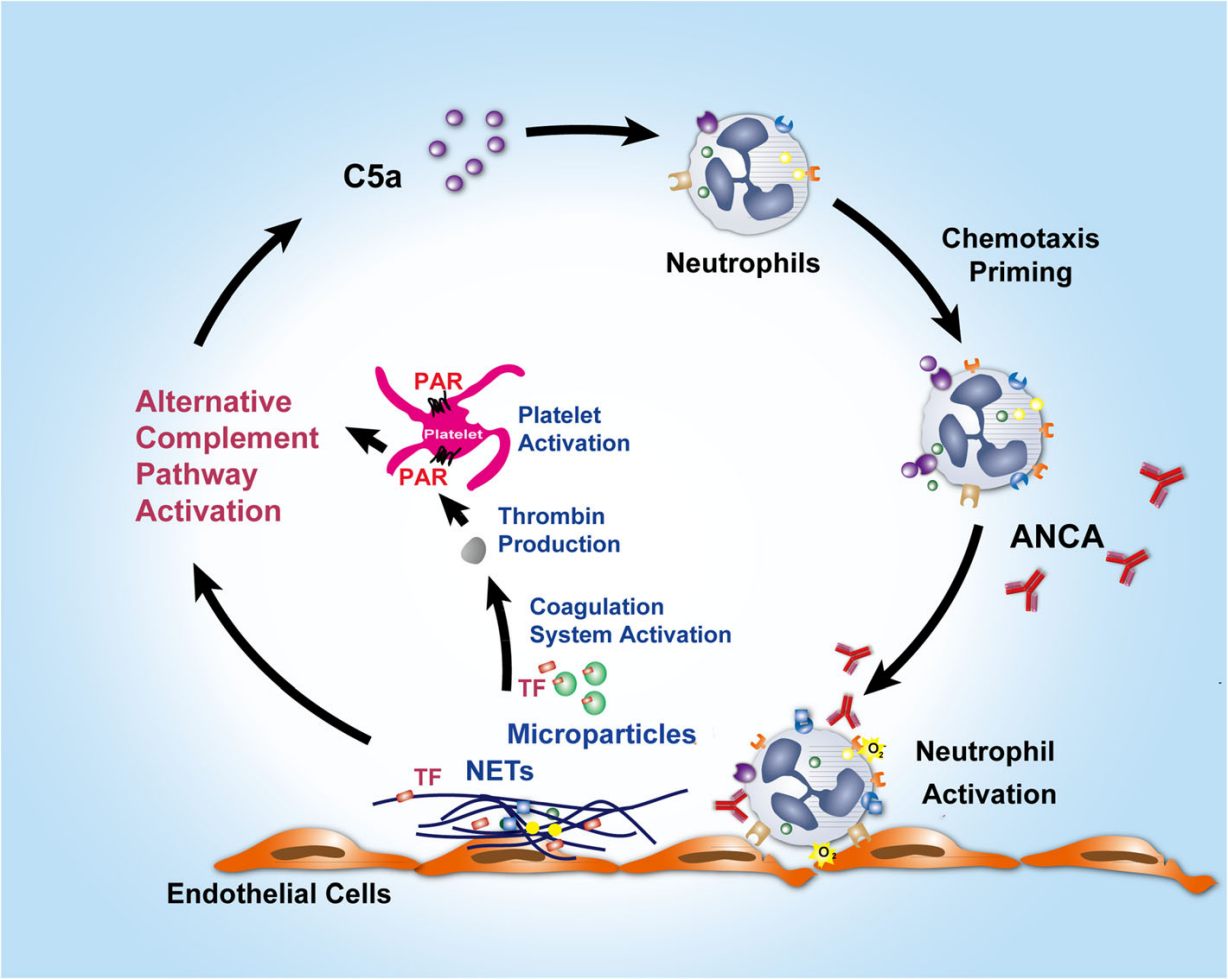
The proposed cross-talk mechanism among ANCA, neutrophils, complement, and platelets in the pathogenesis of AAV. Neutrophil stimulation with cytokines (such as C5a or TNF-α) and ANCA results in respiratory burst and degranulation, which leads to the release of tissue factor (TF)-bearing microparticles and NETs that subsequently activate the coagulation system and generate thrombin. Thrombin can activate platelets through PARs. Such activated platelets can activate the alternative complement pathway. Additionally, activated neutrophils can also activate the alternative complement pathway *via* their cell membranes, microparticles, and NETs. This leads to the generation of more C5a, establishing a self-fueling inflammatory amplification loop leading to the vasculitic injury

Cumulative evidence demonstrated that the ANCA, neutrophil, and the complement alternative pathway positive feedback loop play crucial roles in the pathogenesis of AAV [39, 40]. Our previous study reported that C5a-primed neutrophils activated by ANCAs release tissue factor (TF)-expressing neutrophil microparticles and neutrophil extracellular traps, which subsequently activate the coagulation system and generate thrombin [11]. Our findings in the current study further demonstrate that the thrombin in plasma of AAV patients can activate platelets, and such stimulated platelets can subsequently activate the alternative complement pathway. These emerging functions of platelets bridge neutrophils and the alternative pathway of complement activation, which establish an especially self-fueling inflammatory amplification loop leading to vasculitic injury. The proposed mechanism of the cross-talk among ANCA, neutrophils, complement particles, and platelets in the pathogenesis of AAV is shown in Fig. 5.

## Conclusions

In conclusion, our study reveals that platelets are activated in AAV patients. Such activation is partially attributed to the thrombin-PARs pathway, which may account for the hypercoagulable state in active AAV. Furthermore, activated platelets trigger the alternative complement pathway. These findings support the possible roles of platelets in the pathogenesis of AAV and indicate their potential as novel therapeutic targets.

## Abbreviations

AAV: ANCA-associated vasculitis
ANCA: Antineutrophil cytoplasmic antibody
AZA: Azathioprine
BVAS: Birmingham Vasculitis Activity Score
CTX: Cyclophosphamide
EGPA: Eosinophilic granulomatosis with polyangiits
ELISA: Enzyme-linked immunosorbent assay
ENT: Ear, nose, throat
FACS: Fluorescent-activated cell sorting
GPA: Polyangiitis
GPRP: Gly-Pro-Arg-Pro
MCD: Minimal change disease
MPA: Microscopic polyangiitis
MPO: Myeloperoxidase
PARs: Protease-activated receptors
PPACK: D-Phe-Pro-Arg-CH_2_Cl
PR3: Protinase 3
PRP: Platelet-rich plasma
Scr: Serum creatinine
TF: Tissue factor
TRAP: Thrombin receptor-activating peptide
VTE: Venous thromboembolic events
